# On the Versatility of von Willebrand Factor

**DOI:** 10.4084/MJHID.2013.046

**Published:** 2013-07-10

**Authors:** Antoine Rauch, Nikolett Wohner, Olivier D. Christophe, Cécile V. Denis, Sophie Susen, Peter J. Lenting

**Affiliations:** 1 INSERM Unit 770, Le Kremlin-Bicêtre, France.; 2 UMR_S 770, Univ Paris-Sud, Le Kremlin-Bicêtre, France.; 3 Department of Hematology & Transfusion, Lille University Hospital, Lille, France.; 4 Equipe d’Accueil 2693, Lille-II University, Lille, France.

## Abstract

Von Willebrand factor (VWF) is a large multimeric protein, the function of which has been demonstrated to be pivotal to the haemostatic system. Indeed, quantitative and/or qualitative abnormalities of VWF are associated with the bleeding disorder Von Willebrand disease (VWD). Moreover, increased plasma concentrations of VWF have been linked to an increased risk for thrombotic complications. In the previous decades, many studies have contributed to our understanding of how VWF is connected to the haemostatic system, particularly with regard to structure-function relationships. Interactive sites for important ligands of VWF (such as factor VIII, collagen, glycoprotein Ibα, integrin αIIbβ3 and protease ADAMTS13) have been identified, and mutagenesis studies have confirmed the physiological relevance of the interactions between VWF and these ligands. However, we have also become aware that VWF has a more versatile character than previously thought, given its potential role in various non-hemostatic processes, like intimal thickening, tumor cell apoptosis and inflammatory processes. In the presence review, a summary of our knowledge on VWF structure-function relationships is provided in the context of the “classical” haemostatic task of VWF and in perspective of pathological processes beyond haemostasis.

## Introduction

Von Willebrand factor (VWF) is a protein that has historically been known for its role in the haemostatic process. However, many hurdles had to be overcome between the initial description in 1926 of the bleeding tendency that is now known as Von Willebrand disease (VWD)^1^ and the identification of the protein that is associated with this disorder. Indeed, it took 30 years after the seminal paper by Erik von Willebrand, before it was reported that the bleeding episodes in von Willebrand disease could be corrected upon the infusion of a plasma factor.^2^ The search for the identity of this plasma component was far from simple. Indeed, it was complicated by the multimeric nature of VWF and the notion that VWF circulates in complex with coagulation factor VIII (FVIII), the protein that is associated with hemophilia A. The technical difficulties that needed to be addressed have nicely been put in context in several personal anecdotes describing the events that led to the discovery in the early 1970s that VWF and FVIII are separate proteins and that VWF is a multimeric protein.^3^–^5^ The identification of VWF as a plasma component that is associated with VWD provided the basis for numerous additional studies. For starters, the purified protein was used to determine its sequence, which in turn was needed to clone the gene encoding VWF.^6^–^10^ This breakthrough stimulated the rapid expansion of our knowledge on the epidemiology, genetics and molecular basis of VWD.^11^,^12^ With the help of recent multicenter studies in Europe, Canada and the USA, our insight into the complex genetic background of VWD has been dramatically improved, a necessary step to further refine clinical and laboratory diagnosis of the disease.^13^,^14^ Clinical studies further taught us that the critical role of VWF in haemostasis is not only obvious from the bleeding tendency that is associated with its functional deficiency, but also in view of its relationship with thrombotic disorders. Increased levels of VWF have been shown to be predictive for atherothrombotic complications.^15^–^22^ In line with VWF being a risk factor for atherothrombotic complication, a recent study reported a reduced prevalence of arterial thrombosis in patients with VWD.^23^ Importantly, the role of VWF in the development of thrombotic complications is not limited to myocardial infarctions, but also include stroke and venous thrombosis.^24^–^28^ Of note, the contribution of VWF to venous thrombosis may be both direct as part of a complex with neutrophil extracellular traps (NETs),^29^ and indirect via FVIII (which is an independent risk factor for venous thrombosis), given the role of VWF as a determinant of FVIII plasma levels.^30^,^31^

Fundamental studies on the structure-function relationships of VWF provided insight into how this multimeric protein supports the different aspects of the haemostatic process. Importantly, from these studies it also became clear that VWF has a more versatile character than previously thought, given its potential role in various non-hemostatic processes, like cell proliferation and tumor cell apoptosis.^32^ In the present review, an overview of our current knowledge of VWF structure and function will be provided. Subsequently, we will describe the contribution of VWF to (patho)-physiological processes beyond haemostasis. Finally, we will discuss how shear stress and modulation of multimer size regulate classical and novel functions of VWF.

## Structure of VWF

**Figure 1:**
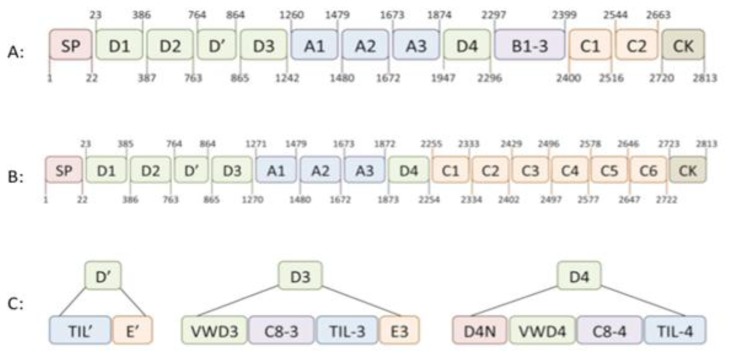
Domain structure of VWF The molecular architecture of VWF is characterized by the presence of distinct domain structures. Panel A represents the arrangement of five different structures according to the original analysis of the VWF sequence (reviewed by Pannekoek & Voorberg).^38^ The numbering of the domain boundaries has been used in our laboratory in the previous years. Panel B shows the domain organization as has recently been proposed by Zhou et al.^41^ One striking difference with the original domain structure is the replacement of the B1-3 - C1 - C2 domain region by 6 homologous C-domains. In addition, their analysis revealed that the D-domains consist of various independent structures, which are highlighted in panel C. The D1, D2 and D3 domains each contain a VW-domain, a trypsin inhibitor-like (TIL)-structure, a C8 fold and an E module. The D′ region lacks the VW domain and TIL-structure. The D4 domain lacks the E module, but instead comprises a unique sequence designated D4N.

The biosynthesis of VWF has been described extensively in several excellent reviews (see for example Wagner^33^ and Sadler^34^,^35^). Its synthesis is limited to endothelial cells and megakaryocytes,^36^,^37^ where it is produced as a single chain pre-pro-protein. It consists of a 22-amino acid signal peptide, a 741-amino acid propeptide and a mature subunit of 2050 amino acids (Figure 1).^33^–^35^ Initial analysis of the VWF primary structure revealed that the molecular architecture of the pro-protein distinguishes a discrete domain structure, arranged as D1-D2-D′-D3-A1-A2-A3-D4-B1-B2-B3-C1-C2-CK, with the propeptide comprising the D1-D2 domains, and the mature subunits the remaining domains.^33^–^35^,^38^ More recently, the domain structure of VWF has been re-evaluated using structural information from other proteins with homologous domains in combination with electron microscopy techniques.^39^–^41^ This exercise revealed a number of interesting insights. First, it allowed a more detailed assignment of disulfide bonds between cysteines throughout the molecule.^41^ Second, it appeared that the D-domains have a more complex structure than initially thought. In fact, D-domains consist of 4 independent structures: a von Willebrand domain, a Cysteine-8 structure, a trypsin-inhibitor-like (TIL)-fold and an E module (Figure 1).^41^ Third, the region carboxyterminal to the D4 domain (i.e. the B1-B2-B3-C1-C2 domains) was recognized to consist of six consecutive C-domains instead of two, with the Arg-Gly-Asp (RGD)-integrin recognition sequence being located in the C4 domain (Figure 1).^41^ These new insights in VWF structure will help us to better understand the cross-talk between domains in the functions of VWF.

A most intriguing aspect of VWF biology concerns the multimeric structure of the protein. The mature VWF protein exists as a heterologous series of covalently-linked mature subunits ranging from dimers (molecular weight 0.5 millionDa) to large polymers consisting of over 40 subunits (molecular weight >20 millionDa).^33^–^35^ As will be discussed later in this review, the multimer structure is important for a subset of VWF functions, and regulation of multimer size and quaternary structure is an important tool to modulate these functions.

## The classical functions of VWF: FVIII binding

The intricate linkage between VWF and FVIII is perhaps best illustrated by the nomenclature that was previously used to distinguish between the coagulation- and platelet-related activities of the complex: FVIII coagulant activity (FVIII:C), FVIII related antigen (FVIII:RAg) and FVIII ristocetin cofactor activity (FVIII:RCF).^42^ In fact, some still use the term FVIII:RAg instead of VWF to describe staining of endothelial cells in the immunohistochemical analysis of healthy and pathological tissues (see *e.g.* Bauer *et al.*^43^).

VWF and FVIII circulate in a tight non-covalent complex in the circulation, and the affinity is estimated to be less than 1 nM.^44^,^45^ The binding site for FVIII is located in the amino-terminal D’D3 region, spanning residues 764–1035.^46^,^47^ In a recent study, Castro-Nunez and coworkers used an approach of mass spectrometer-assisted footprinting to discover that VWF residues Ser-764 and Lys-773 seem to be directly involved in the binding of FVIII.^48^ The complementary binding site in FVIII has also been identified, involving residues at both the amino- and carboxyterminal regions of the FVIII light chain.^49^,^50^

The physiological relevance of VWF/FVIII complex formation is exemplified by the markedly reduced FVIII plasma levels in patients with undetectable VWF levels (VWD-type 3) or with a defect in the FVIII-interactive site of VWF (VWD-type 2N).^51^–^53^ Indeed, the majority of VWD-type 2N mutations are located in the region spanning residues 764–1035,^54^ suggesting that these mutations affect FVIII binding directly by modulation of the FVIII interactive site.

VWF protective function towards FVIII is related to several aspects:^55^ (1) VWF stabilizes the heterodimeric structure of FVIII;^56^ (2) VWF protects FVIII from proteolytic degradation by phospholipid-binding proteases like activated protein C and activated factor X (FXa);^57^,^58^ (3) VWF interferes with binding of FVIII to negatively-charged phospholipid surfaces, which are for example exposed on activated platelets;^45^ (4) VWF inhibits binding of FVIII to activated factor IX (FIXa), thereby denying FVIII access to the FX-activating complex;^59^ (5) VWF shields FVIII from part of the inhibitory antibodies that may be generated during FVIII-replacement therapy in about 30% of the severe hemophilia A patients;^60^–^63^ and (6) VWF prevents the uptake of FVIII by some cells, including dendritic cells.^64^,^65^ In view of the role of dendritic cells in antigen-presentation to T-cells, this latter function may be of relevance regarding the immune-response towards FVIII that has been observed in the treatment of hemophilia patients. In several *in vivo* studies using mice, it has been shown that the addition of VWF reduces the immune-response towards FVIII.^63^,^66^–^68^ This may suggest that the presence of VWF in therapeutic FVIII preparations may influence the development of inhibitory antibodies, although epidemiological studies have revealed conflicting data on this possibility.^69^–^73^

Apart from its protective role, VWF may also play a role in the targeting of FVIII to sites of vascular injury.^74^ It should be noted that complex formation is not an absolute requirement for FVIII to reach the developing thrombus, as has been shown in studies using VWF-deficient mice.^75^,^76^

## The classical functions of VWF: collagen binding

Shortly after the identification of VWF as a plasma protein, its capacity to adsorb to collagens was reported.^77^ Subsequent studies revealed that a dominant binding site for collagen in VWF is located in the VWF A3 domain involving a discontinuous epitope.^78^–^81^ The A3 domain is able to interact with various types of collagen, including collagens I & III, and the complementary binding sequences in collagen I and III have been deciphered in detail.^82^–^85^ The importance of the A3 domain in binding to collagen is supported by the finding that mutations in or around the collagen binding site may be associated with an increased bleeding tendency.^86^–^89^

An alternative binding-site for collagen in the VWF protein is located in the A1 domain, as has been demonstrated by various research groups.^83^,^90^–^93^ However, opposite findings have been reported concerning the contribution of the A1-domain in facilitating VWF-platelet interactions under conditions of flow.^79^,^94^ Nevertheless, it appears that some mutations in the A1 domain found in VWD patients may affect collagen binding, providing a rationale for the bleeding tendency in these patients.^95^–^98^

It should be noted that the bleeding tendency associated with mutations in the collagen binding site is usually mild, which is in line with the observation that a murine VWF variant with a defective collagen binding in the A3 domain displays no defect in the correction of the bleeding time in a tail clip-model for normal haemostasis.^99^,^100^ In contrast, this mutant shows a strongly delayed occlusion time in a ferric chloride-induced model of vascular injury, suggesting that blocking VWF-collagen interactions could be a potential therapeutic approach in the treatment of arterial thrombosis.^100^ This possibility has been explored in animal models for thrombosis, revealing that antibodies blocking VWF-collagen interactions are efficient in reducing the thrombotic tendency.^101^,^102^ Many *in vitro* studies revealed that VWF-collagen interactions are needed for the recruitment of platelets particularly under conditions of high shear rates (for reviews see Sixma *et al.*^103^ and Nuyttens *et al.*^104^). In spite of this important function, defects in collagen binding are associated with only a mild bleeding tendency. The explanation for this apparent contradiction may originate from the redundancy in the process that mediates platelets adheres to collagen. First, VWF contains multiple collagen-binding sites, which may perhaps compensate for each other under particular conditions. Second, platelets contain other collagen receptors, such as α2β1 and Glycoprotein-VI (GpVI), that could allow them to interact with collagen in the absence of VWF.^105^ It should be mentioned that both receptors do not resist high shear forces,^105^ indicating that they are unable to function as a back-up system for VWF under high shear conditions. Finally, the subendothelial matrix comprises also other components that function as an adhesive surface for VWF, such as tenascin-C and laminin.^106^,^107^ However, the binding sites for these proteins have not yet been identified.

From a structural point of view, the multimeric VWF protein attached to the collagen surface will undergo shear stress-induced conformational changes that lead to the exposure of the binding site for its platelet-receptor GpIb.^108^ Interestingly, binding to collagen has also a secondary effect, in that it results in release of FVIII from the VWF molecule. This phenomenon was already recognized in the original manuscript that described the adsorption of VWF to collagen,^77^ and was further elaborated by Bendetowicz and colleagues.^109^ The reason for this release is yet unclear, but it could be that release of FVIII from collagen-bound VWF makes it more rapidly available for the coagulation cascade: VWF-bound FVIII is poorly activated by FXa/phospholipids, whereas VWF-free FVIII is efficiently activated by this complex.^57^ Alternatively, this collagen-induced release could be a mechanism to prevent FVIII binding to VWF that is located in the subendothelial matrix, preventing undesired extravasation of FVIII.^109^

## The classical functions of VWF: platelet binding

A key function of VWF is to mediate the recruitment of platelets to sites of vascular injury, especially at those locations where collagen-binding platelet-receptors do not resist high shear forces. Interactions between platelets and VWF are mediated by two distinct platelet receptors: GpIbα and integrin αIIbβ3. GpIbα is part of the GpIb-IX-V complex that is abundantly expressed at the platelet surface.^110^,^111^ Contacts between VWF and GpIbα require the VWF A1 domain, and the GpIbα interactive site has been elucidated at the atomic level using co-crystal structures of the VWF A1 domain and a soluble GpIbα fragment.^112^–^114^ Mutations of residues in the VWF A1 domain that cover the interactive surface with GpIbα have indeed found to be associated with impaired VWF function and a bleeding tendency in patients with VWD-type 2M.^12^ The VWF-GpIbα interaction is probably the best-studied aspect of VWF at both the functional and structural level, and its importance for the formation of platelet-rich thrombi has been extensively reviewed elsewhere (see for instance references 108,115–117).

The binding site for αIIbβ3 is located in the C1 domain of VWF (C4 domain according to the new annotation proposed by Zhou *et al*.^41^) and involves the classical Arg-Gly-Asp (RGD) recognition sequence for integrins. The function of the VWF-αIIbβ3 interaction is related to the enforcement of platelet-platelet interactions as has been demonstrated in several in vitro studies.^118^–^121^ However, since several other ligands (notably fibrinogen) are capable of doing so as well, this VWF function has long been thought to be redundant. This view is compatible with the notion that so far no patients having mutations in the αIIbβ3 binding sequence have been reported. However, studies using a mouse model expressing a VWF mutant with defective αIIbβ3 binding have forced us to change this view. Although mice expressing this mutant show normal correction of the bleeding time in a tail clip-model for haemostasis, they are characterized by an impaired vessel occlusion time in a ferric chloride-induced model of vascular injury.^99^,^100^ A similar reduction of vessel occlusion was observed in mice treated with antibodies against the RGD-sequence of VWF.^102^ More detailed analysis of thrombus formation in these mice revealed that initial thrombus formation is unaffected. However, larger thrombi seem to dissolve as a result of the increased hydrodynamic forces to which the growing thrombus is exposed.^100^,^102^ This strongly suggests that the VWF-αIIbβ3 interaction is not redundant, but of physiological relevance with regard to the stabilization of the growing thrombus.

## Novel aspects of VWF function: the molecular bus

As described in the paragraph “The classical functions of VWF: FVIII binding”, VWF is particularly known as a carrier protein for FVIII in the circulation to maintain appropriate FVIII plasma levels. However, in recent years it has become clear that FVIII is not the only protein that circulates in complex with VWF in the circulation. Other examples of proteins that are associated with VWF in plasma include ADAMTS13,^122^,^123^ osteoprotegerin,^124^–^126^ angiopoietin-2 (Christophe OD, Cherel G, Lenting PJ, Denis CV; unpublished publications) and two members of the galectin family, galectin-1 and galectin-3.^127^ It would not be surprising if this list of VWF-bound proteins will grow in the future. For instance, Turner & Moake recently published that several members of the complement family (*i.e.* C3, C5 and factors B, D, P H & I) attach to VWF that is freshly released from endothelial cells.^128^ It seems reasonable to assume that at least some of these proteins remain associated to VWF upon release from the endothelial surface into the circulation.

Like for FVIII, galectin-1 and galectin-3 plasma levels were higher in wild-type mice compared to mice deficient for VWF,^127^,^129^ suggesting that VWF is needed to stabilize galectin-1 and galectin-3 in the circulation. With regard to osteoprotegerin, a recent study revealed a positive correlation between VWF and osteoprotegerin levels in a cohort consisting of patients with cardiovascular disease and asymptomatic controls.^126^ This correlation appeared particularly relevant in asymptomatic individuals without coronary calcification. These recent findings might suggest that VWF could play a similar protective role to stabilize osteoprotegerin in plasma. Of course, additional studies are needed to support this point of view. An opposite observation has been made regarding ADAMTS13 in that an inverse relationship between plasma levels of VWF and ADAMTS13 was reported.^130^ In addition, ADAMTS13 levels were ~40% higher in patients lacking circulating VWF than in control individuals.^130^ How VWF influences ADAMTS13 plasma levels remains to be determined. One possible explanation can be that VWF-bound ADAMTS13 is cleared in *conjunction* with VWF, which has a shorter half-life than ADAMTS13.^131^,^132^

The wide variety of proteins that are bound to VWF in the circulation raises a number of questions. First, how many passengers can be on the VWF bus at the same time? For FVIII and both galectins, we know that their plasma concentrations are about 100-fold lower than that of VWF, which suggests that they will not occupy all the places that are available. As for ADAMTS13, Feys *et al.* calculated that it circulates in complex with VWF in a stoichiometry of 1:250, also indicating that the majority of the VWF subunits remain non-occupied.^123^ A second question is: what are the functional consequences of complex formation? VWF protects FVIII and may promote its targeting to sites of vascular injury. In contrast, FVIII may have the opposite effect on VWF, as it has been reported that the presence of FVIII promotes VWF degradation by ADAMTS13.^133^ With regard to the galectins, angiopoietin-2 and osteoprotegerin, the functional consequences of their binding to VWF have been investigated to a limited extent, if at all. In view of the large size of the VWF protein, it seems conceivable that VWF has a profound effect of the functionality of these proteins in that it may prevent the interaction with their natural ligand via sterical hindrance. However, many unknowns remain in this respect, and it would be of interest to explore the mutual functional effects between VWF and its passengers.

## Novel aspects of VWF function: cell effector in the angiogenic process

**Figure 2:**
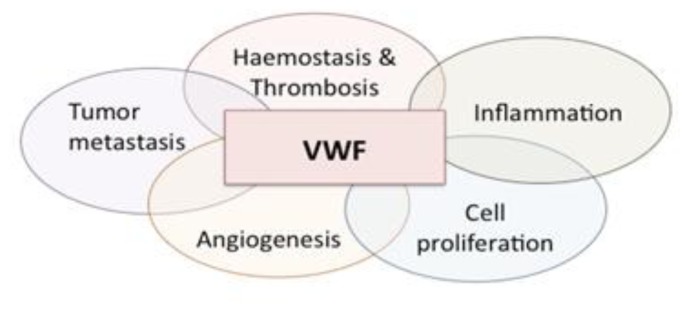
The functional diversity of VWF VWF is best known for its link with the hemostatic system, where it participates in the recruitment of platelets to the injured vessel wall and acts as a carrier protein for FVIII. The physiological relevance of this function is underscored by VWF being associated with bleeding problems (VWD and acquired VW syndrome) as well as thrombotic complications (myocard infarction, stroke and venous thrombosis). More recently it has been found that VWF is involved in other patho-physiological processes as well, such as tumor metastasis (inducing tumor cell death), angiogenesis (which could provide a rationale for the relatively frequent occurrence of angiodysplasia in VWD patients), cell proliferation (associated with enhanced intima thickening after angioplasty and in CADASIL), and inflammatory processes (as observed in animal models for atherosclerosis, stroke, wound healing and experimental allergic encephalomyelitis).

During the last two decades, more than 20 proteins have been identified that interact with VWF, several of them being involved in cellular signaling processes.^32^ Consequently, VWF has been linked to other (patho)physological processes than haemostasis as well, including angiodysplasia, tumor metastasis and smooth muscle cell proliferation (Figure 2; for recent reviews on these topics see references 32,134,135). However, the mechanism by which VWF is linked to this processes is largely undefined. For each of the three conditions a brief overview of our current knowledge will be provided.

With regard to the angiogenic process, it has been found that the absence of VWF increases endothelial cell proliferation *in vitro*.^136^ In line with this observation, VWF-deficient mice display an increased vessel density of the vasculature in the ears in comparison to VWF-expressing mice,^136^ suggesting that VWF acts as a negative modulator of angiogenesis. The molecular basis of this modulatory effect is yet unclear. Results from the study by Starke and colleagues point to an effect of VWF on vascular endothelial growth factor (VEGF)-dependent angiogenesis, which proceeds via multiple intracellular and extracellular pathways dependent on αVβ3 and angiopoietin-2.^136^ Given that both proteins are ligands for VWF, it seems possible that VWF acts on the angiogenic process via interactions with both proteins. However, the endothelial cells contain several other VWF-binding proteins with pro- and anti-angiogenic properties, such as galectins-1 and -3,^137^,^138^ connective tissue growth factor^139^ and insulin-like growth factor binding protein-7.^140^ This points to a complex role of VWF, able to affect the angiogenic process at different levels.

Irrespective of the precise mechanism, the link between VWF and angiogenesis seems to be of physiological relevance, given the relatively frequent occurrence of angiodysplasia in patients with VWD.^134^,^141^ Angiodysplasia is characterized by vascular malformations resulting from an impaired angiogenic process, and is often clinically manifested via gastro-intestinal bleedings.^142^ Interestingly, the manifestation of angiodysplasia in VWD patients is observed more frequently in patients that lack high multimers, either because of hereditary defects^141^,^143^ or because of acquired conditions, such as Heyde’s syndrome or patients carrying circulatory assist devices.^144^,^145^ Why there is this specific link with high molecular weight multimers is unclear. Perhaps it involves a mechanism that is similar to the interaction between VWF and GpIbα, which also is more efficient for the larger multimers compared to smaller variants. The possibility exists that VWF interacts in a multimer size-dependent manner with so far unidentified cellular receptors (expressed on endothelial cells or other cells in the vascular wall) that are involved in maintaining the vascular integrity. Solving this enigma would be of interest for the development of novel therapeutic means to manage this severe complication of VWD.

## Novel aspects of VWF function: cell effector in smooth muscle cell proliferation

Care should be taken in extrapolating the anti-proliferative effect of VWF towards VEGF-stimulated endothelial cells also to other cell types. As will be discussed in this section, VWF may also exert a proliferative effect, demonstrating that the cell effector function of VWF may be very much dependent on the local cellular environment. Upon damage of the vascular endothelial layer, VWF is able to penetrate into the intima of large peripheral vessels, where it is exposed to smooth muscle cells.^146^–^148^ The deposition of VWF in the intima coincides with intimal thickening,^149^ suggesting that VWF plays a role in the pathogenesis of intimal hyperplasia by promoting smooth muscle cell proliferation. This possibility is supported by *in vitro* experiments showing that VWF directly stimulates smooth muscle cell proliferation,^149^ The transcriptional changes in smooth muscle cells that are being induced upon exposure to VWF have recently been unraveled, and involve multiple genes associated with growth factor stimulation.^150^

The effect of VWF-dependent smooth muscle cell proliferation is not only of relevance with regard to vascular damage, for instance as a consequence of an angioplasty procedure,^146^,^147^ but may also be of importance in view of the hereditary disorder CADASIL (cerebral autosomal dominant arteriopathy with subcordial infarcts and leukoencephalopathy).^150^ The clinical phenotype of this disorder includes recurrent strokes and dementia. Analysis of brain sections of CADASIL-patients revealed that VWF is abundantly present in the brain vessels, particularly in the subarachnoid arteries that are characterized by concentric thickening of the media and adventitia.^150^ The identification of VWF as a player in CADASIL-related smooth muscle cell proliferation could provide the basis for a novel therapeutic approach in the treatment of these patients.

## Novel aspects of VWF function: cell effector in apoptosis

The versatility of VWF is nicely illustrated by the notion that VWF is not only capable of stimulating cell proliferation but also by its capacity to induce cell death. Again, it is important to take into account the local cellular environment in this regard, since the apoptotic function of VWF is probably restricted to but a few cell types. First, it was shown that VWF is able to induce platelet apoptosis via interactions with GpIbα, thereby initiating the caspase-3, Bak and Bax-dependent apoptosis pathway.^151^ The physiological consequences of this finding remain to be determined, but they could be of relevance for those conditions where there are enhanced VWF-platelet interactions without the need for thrombus formation. One such a condition could be VWD-type 2B, where gain-of-function mutations in the VWF A1 domain result in spontaneous VWF-platelet interactions.

Tumor cells are another cell type that might be susceptible to VWF-induced apoptosis. Unexpectedly, tumor cells were found to have a higher metastatic potential in VWF-deficient mice than in VWF-expressing control mice.^152^ This higher metastatic potential appeared to be the result of a longer survival of living cells in the absence of VWF.^152^*In vitro* studies confirmed that VWF induced death of tumor cells.^152^,^153^ The underlying mechanism of VWF-induced cell death remains unclear, although the observation that VWF-tumor cell interactions were mediated by αVβ3 suggest that VWF induces cell death via this integrin.^152^ The capacity of VWF to induce tumor cell death in an αVβ3-dependent fashion was recently confirmed in an elegant study by Mochizuki and colleagues.^154^ However, they also identified a series of tumor cells that was capable of escaping VWF-induced cell death. The explanation for this resistance against VWF-induced apoptosis was rather unexpected: they found that tumor cells are able to secrete a protease (ADAM-28) that is able to degrade VWF.^154^ Thus, VWF negatively regulates tumor cell survival, and certain tumor cells have armed themselves against VWF via the production of a protease that destroys the pro-apoptotic function of VWF.

## Novel aspects of VWF function: a pro-inflammatory agent

The adhesive nature of the VWF protein allows it to function as a landing platform for platelets. This raises the question whether this adhesive capacity is selective for platelets, or whether also other cells are able to adhere VWF. We have previously addressed this issue, and observed that leukocytes may adhere to immobilized VWF under conditions of low shear.^155^ In the same study, we were able to identify PSGL-1 and β2-integrins as potential counter-receptors for VWF at the leukocyte surface.^155^ More recently, we also identified Siglec-5 as a leukocyte receptor that is able to interact with VWF, although we did not test whether Siglec-5 contributes to leukocyte-VWF interactions under conditions of flow.^156^ Evidence is also accumulating from other studies that VWF may actively participate in leukocyte recruitment. First, platelet-decorated VWF strings at the cellular surface efficiently attract leukocytes, even under conditions of high shear stress.^157^ Furthermore, VWF-platelet complexes play a crucial role in the extravasation of leukocytes upon an inflammatory response.^158^

The participation of VWF in the inflammatory response has been confirmed in several animal models for inflammation, such as atherosclerosis, wound healing, experimental allergic encephalomyelitis, and stroke.^159^–^162^ Whether VWF plays a similar important role in the human pathology of these diseases is unclear, which could be related to the multi-factorial nature of such inflammatory conditions. For instance, VWF-deficient mice and pigs develop fewer atherosclerotic lesions compared to VWF-expressing animals, suggesting that VWF could participate in attracting leukocytes to lesion sites.^161^,^163^ However, human studies revealed conflicting information whether or not atherosclerosis is reduced in patients with VWD (recently reviewed by van Galen *et al.*^164^). One crucial difference that could explain the observed differences between humans and animals that lack VWF is that patients receive replacement therapy to replenish the reservoir of circulating VWF. As such they are less deficient in VWF compared to the animals.

## Regulation of classic and new VWF functions: multimer size and shear stress

In the circulation, VWF is exposed to many of its ligands, including platelets. Therefore, mechanisms need to be in place to prevent premature interactions between VWF and platelets in order to prevent undesired vessel occlusion.^165^ On the other hand, for some ligands (such as FVIII) it is actually necessary that VWF is able to interact with them in a constitutive manner, without a strict regulation. From these two examples it becomes clear that the versatility of VWF is not only restricted to its functions, but also with regard to the regulation of these functions.

There are two dominant mechanisms in place that contribute to the regulation of VWF function. First, VWF is able to change conformation in response to shear stress.^166^–^168^ In the normal circulation VWF is present as a globular protein, whereas exposure to increased shear forces drives the protein into an elongated conformation.^166^–^168^ This change in conformation has a number of consequences:^169^ (1) it results in decryption of the GpIbα binding site, allowing platelet binding;^108^ (2) the cleavage site for ADAMTS13 becomes available;^170^,^171^ (3) it exposes methionine residues that are sensitive to oxidation;^172^ (4) it promotes disulfide bridge formation between cysteine-residues in the CK-domain;^173^,^174^ (5) it enhances VWF self-association;^175^ and (6) it turns VWF into a ligand for its clearance receptor LRP1.^176^ In contrast to these shear stress-dependent phenomenon, the interactions between VWF and collagen or FVIII do not seem to require shear stress-induced conformations, as they already occur under static conditions.

Do these shear stress-induced conformational changes also affect the novel functions of VWF? In most cases, this does not seem to be the case. The effects of VWF on angiogenesis, smooth muscle cell proliferation and tumor cell death have usually been investigated *in vitro* under static conditions. Of course, this does not necessarily mean that shear stress will not affect these functions. However, additional studies are needed to get insight into the role of shear stress on novel VWF functions.

A second mechanism to regulate VWF function is to vary its multimer size, and several mechanisms are at hand to do so. One protein that contributes to the regulation of VWF multimer size is thrombospondin, which controls VWF multimer size via the introduction of new thiols.^177^ Second, shear stress-induced self-association may contribute to enlarge the multimer size of VWF.^175^ However, the most dominant regulator seems to be ADAMTS13, which is able to proteolytically degrade VWF via cleavages in the A2 domain between residues Tyr1605 and Met1606.^178^ The mechanism by which ADAMTS13 recognizes and cleaves its substrate has been described in detail in an excellent review by Crawley and colleagues.^179^ The importance of ADAMTS13 in the regulation of VWF multimer size in view of its hemostatic properties is evident from the thrombotic complications that occur in the absence of ADAMTS13, a disorder known as thrombotic thrombocytopenic purpura.^180^–^182^ However, does ADAMTS13 also affect non-hemostatic functions of VWF? There are indications that this is indeed conceivable. First, we already mentioned that angiodyplasia is particularly associated with VWD patients that lack high molecular weight multimers, such as in VWD-type 2A.^143^ Apparently, an increased degradation of VWF interferes with the property of VWF to maintain the integrity of the vasculature. Second, increased leukocyte rolling on unstimulated veins and increased leukocyte adhesion in inflamed veins has been observed in mice deficient for ADAMTS13. Moreover, it has been found that the absence of ADAMTS13 exacerbates the inflammatory response in animal models for stroke and atherosclerosis.^159^,^183^–^185^ Apparently, proteolytic degradation of VWF by ADAMTS13 downregulates the inflammatory potential of VWF. With regard to the effect of VWF on tumor cell death, the importance of multimer size is yet unclear. It should be noted that ADAM-28 reduced VWF multimer size via proteolysis at two distinct sites in the VWF protein, which coincides with a loss in apoptotic potential.^154^ Since these sites are located away from the αVβ3-recognition sequence (*i.e.* the RGD-motif), it seems conceivable that VWF multimer size plays a role in the interaction with tumor cells to initiate the apoptotic process.

## Conclusion

Forty years after its first purification from plasma, VWF still carries many mysteries. Its versatility is steadily being exposed but even its role in thrombosis, once thought to be well understood, is still eluding us. Indeed, the notion that a VWF-mutant unable to bind αIIbβ3 is protective against thrombosis in a ferric chloride-induced model for arterial thrombosis while it is without effect in a stroke model, is a perfect example of this constant reassessment that is forced upon us.^100^,^186^ The possibility to target VWF in the management of thrombotic disorders should therefore be considered as a real option. With regard to the non-hemostasis functions of VWF, many avenues also remain to be explored. The combination of data originating from both clinical and basic studies on VWF will no doubt be instrumental in expanding our knowledge of this intriguing protein.

## References

[1] Willebrand EA (1931). Über hereditäre Pseudohämophilie. Act Med Scand.

[2] Nilsson IM, Blomback M, Jorpes E, Blomback B, Johansson Sv (1957). Willebrand’s Disease and its Correction with Human Plasma Fraction 1-0. Act Med Scand.

[3] Owen WG (2005). Big piece, little piece or: yes, factor VIII is a protein. J Thromb Haemostas.

[4] Bouma BN, Van Mourik JA (2006). Unraveling the mystery of von Willebrand factor. J Thromb Haemostas.

[5] Ruggeri ZM (2007). Von Willebrand factor: looking back and looking forward. Thromb Haemost.

[6] Ginsburg D, Handin RI, Bonthron DT (1985). Human von Willebrand factor (vWF): isolation of complementary DNA (cDNA) clones and chromosomal localization. Science.

[7] Lynch DC, Zimmerman TS, Collins CJ (1985). Molecular cloning of cDNA for human von Willebrand factor: authentication by a new method. Cell.

[8] Verweij CL, Diergaarde PJ, Hart M, Pannekoek H (1986). Full-length von Willebrand factor (vWF) cDNA encodes a highly repetitive protein considerably larger than the mature vWF subunit. EMBO J.

[9] Sadler JE, Shelton-Inloes BB, Sorace JM, Harlan JM, Titani K, Davie EW (1985). Cloning and characterization of two cDNAs coding for human von Willebrand factor. Proc Natl Acad Sci U S A.

[10] Titani K, Kumar S, Takio K (1986). Amino acid sequence of human von Willebrand factor. Biochemistry.

[11] James PD, Lillicrap D (2012). von Willebrand disease: clinical and laboratory lessons learned from the large von Willebrand disease studies. Am J Hematol.

[12] Goodeve AC (2010). The genetic basis of von Willebrand disease. Blood Rev.

[13] Schneppenheim R, Budde U (2011). von Willebrand factor: the complex molecular genetics of a multidomain and multifunctional protein. J Thromb Haemost.

[14] Sadler JE (2009). Low von Willebrand factor: sometimes a risk factor and sometimes a disease. Hematology Am Soc Hematol Educ Program.

[15] Margulis T, David M, Maor N (1986). The von Willebrand factor in myocardial infarction and unstable angina: a kinetic study. Thromb Haemost.

[16] Thompson SG, Kienast J, Pyke SD, Haverkate F, van de Loo JC (1995). Hemostatic factors and the risk of myocardial infarction or sudden death in patients with angina pectoris. European Concerted Action on Thrombosis and Disabilities Angina Pectoris Study Group. N Engl J Med.

[17] Montalescot G, Philippe F, Ankri A (1998). Early increase of von Willebrand factor predicts adverse outcome in unstable coronary artery disease: beneficial effects of enoxaparin. French Investigators of the ESSENCE Trial. Circulation.

[18] Folsom AR, Rosamond WD, Shahar E (1999). Prospective study of markers of hemostatic function with risk of ischemic stroke. The Atherosclerosis Risk in Communities (ARIC) Study Investigators. Circulation.

[19] Whincup PH, Danesh J, Walker M (2002). von Willebrand factor and coronary heart disease: prospective study and meta-analysis. Eur Heart J.

[20] Morange PE, Simon C, Alessi MC (2004). Endothelial cell markers and the risk of coronary heart disease: the Prospective Epidemiological Study of Myocardial Infarction (PRIME) study. Circulation.

[21] Danesh J, Wheeler JG, Hirschfield GM (2004). C-reactive protein and other circulating markers of inflammation in the prediction of coronary heart disease. N Engl J Med.

[22] Spiel AO, Gilbert JC, Jilma B (2008). von Willebrand factor in cardiovascular disease: focus on acute coronary syndromes. Circulation.

[23] Sanders YV, Eikenboom J, de Wee EM (2013). Reduced prevalence of arterial thrombosis in von Willebrand disease. J Thromb Haemostas.

[24] Wieberdink RG, van Schie MC, Koudstaal PJ (2010). High von Willebrand factor levels increase the risk of stroke: the Rotterdam study. Stroke.

[25] van Schie MC, Wieberdink RG, Koudstaal PJ (2012). Genetic Determinants of Von Willebrand Factor Plasma Levels and the Risk of Stroke: The Rotterdam Study. J Thromb Haemost.

[26] Tsai AW, Cushman M, Rosamond WD (2002). Coagulation factors, inflammation markers, and venous thromboembolism: the longitudinal investigation of thromboembolism etiology (LITE). Am J Med.

[27] Lijfering WM, Rosendaal FR, Cannegieter SC (2010). Risk factors for venous thrombosis - current understanding from an epidemiological point of view. Br J Haematol.

[28] Smith NL, Rice KM, Bovill EG (2011). Genetic variation associated with plasma von Willebrand factor levels and the risk of incident venous thrombosis. Blood.

[29] Fuchs TA, Brill A, Wagner DD (2012). Neutrophil Extracellular Trap (NET) Impact on Deep Vein Thrombosis. Arterioscler Thromb Vasc Biol.

[30] Koster T, Blann AD, Briet E, Vandenbroucke JP, Rosendaal FR (1995). Role of clotting factor VIII in effect of von Willebrand factor on occurrence of deep-vein thrombosis. Lancet.

[31] Campos M, Buchanan A, Yu F (2012). Influence of single nucleotide polymorphisms in factor VIII and von Willebrand factor genes on plasma factor VIII activity: the ARIC Study. Blood.

[32] Lenting PJ, Casari C, Christophe OD, Denis CV (2012). von Willebrand factor: the old, the new and the unknown. J Thromb Haemost.

[33] Wagner DD (1990). Cell biology of von Willebrand factor. Annu Rev Cell Biol.

[34] Sadler JE (1998). Biochemistry and genetics of von Willebrand factor. Annu Rev Biochem.

[35] Sadler JE (2009). von Willebrand factor assembly and secretion. J Thromb Haemost.

[36] Wagner DD, Marder VJ (1983). Biosynthesis of von Willebrand protein by human endothelial cells. Identification of a large precursor polypeptide chain. J Biol Chem.

[37] Sporn LA, Chavin SI, Marder VJ, Wagner DD (1985). Biosynthesis of von Willebrand protein by human megakaryocytes. J Clin Invest.

[38] Pannekoek H, Voorberg J (1989). Molecular cloning, expression and assembly of multimeric von Willebrand factor. Baillieres Clin Haematol.

[39] Springer TA (2011). Biology and physics of von Willebrand factor concatamers. J Thromb Haemost.

[40] Zhou YF, Eng ET, Nishida N, Lu C, Walz T, Springer TA (2011). A pH-regulated dimeric bouquet in the structure of von Willebrand factor. EMBO J.

[41] Zhou YF, Eng ET, Zhu J, Lu C, Walz T, Springer TA (2012). Sequence and structure relationships within von Willebrand factor. Blood.

[42] Johnson SS, Montgomery RR, Hathaway WE (1981). Newborn factor VIII complex: elevated activities in term infants and alterations in electrophoretic mobility related to illness and activated coagulation. Br J Haematol.

[43] Bauer JL, Miklos AZ, Thompson LD (2012). Parotid gland solitary fibrous tumor: a case report and clinicopathologic review of 22 cases from the literature. Head and neck pathology.

[44] Leyte A, Verbeet MP, Brodniewicz-Proba T, Van Mourik JA, Mertens K (1989). The interaction between human blood-coagulation factor VIII and von Willebrand factor. Characterization of a high-affinity binding site on factor VIII. Biochem J.

[45] Nesheim M, Pittman DD, Giles AR (1991). The effect of plasma von Willebrand factor on the binding of human factor VIII to thrombin-activated human platelets. J Biol Chem.

[46] Foster PA, Fulcher CA, Marti T, Titani K, Zimmerman TS (1987). A major factor VIII binding domain resides within the amino-terminal 272 amino acid residues of von Willebrand factor. J Biol Chem.

[47] Takahashi Y, Kalafatis M, Girma JP, Sewerin K, Andersson LO, Meyer D (1987). Localization of a factor VIII binding domain on a 34 kilodalton fragment of the N-terminal portion of von Willebrand factor. Blood.

[48] Castro-Nunez L, Bloem E, Boon-Spijker MG (2013). Distinct roles of Ser-764 and Lys-773 at the N terminus of von Willebrand factor in complex assembly with coagulation factor VIII. J Biol Chem.

[49] Leyte A, van Schijndel HB, Niehrs C (1991). Sulfation of Tyr1680 of human blood coagulation factor VIII is essential for the interaction of factor VIII with von Willebrand factor. J Biol Chem.

[50] Saenko EL, Scandella D (1997). The acidic region of the factor VIII light chain and the C2 domain together form the high affinity binding site for von willebrand factor. J Biol Chem.

[51] Tuddenham EG, Lane RS, Rotblat F (1982). Response to infusions of polyelectrolyte fractionated human factor VIII concentrate in human haemophilia A and von Willebrand’s disease. Br J Haematol.

[52] Morfini M, Mannucci PM, Tenconi PM (1993). Pharmacokinetics of monoclonally-purified and recombinant factor VIII in patients with severe von Willebrand disease. Thromb Haemost.

[53] Nishino M, Girma JP, Rothschild C, Fressinaud E, Meyer D (1989). New variant of von Willebrand disease with defective binding to factor VIII. Blood.

[54] Jacquemin M (2009). Factor VIII-von Willebrand factor binding defects in autosomal recessive von Willebrand disease type Normandy and in mild hemophilia A. New insights into factor VIII-von Willebrand factor interactions. Acta haematologica.

[55] Lenting PJ, van Schooten CJ, Denis CV (2007). Clearance mechanisms of von Willebrand factor and factor VIII. J Thromb Haemost.

[56] Weiss HJ, Sussman II, Hoyer LW (1977). Stabilization of factor VIII in plasma by the von Willebrand factor. Studies on posttransfusion and dissociated factor VIII and in patients with von Willebrand’s disease. J Clin Invest.

[57] Koedam JA, Hamer RJ, Beeser-Visser NH, Bouma BN, Sixma JJ (1990). The effect of von Willebrand factor on activation of factor VIII by factor Xa. Eur J Biochem/FEBS.

[58] Fay PJ, Coumans JV, Walker FJ (1991). von Willebrand factor mediates protection of factor VIII from activated protein C-catalyzed inactivation. J Biol Chem.

[59] Lenting PJ, Donath MJ, van Mourik JA, Mertens K (1994). Identification of a binding site for blood coagulation factor IXa on the light chain of human factor VIII. J Biol Chem.

[60] Suzuki T, Arai M, Amano K, Kagawa K, Fukutake K (1996). Factor VIII inhibitor antibodies with C2 domain specificity are less inhibitory to factor VIII complexed with von Willebrand factor. Thromb Haemostas.

[61] Amano K, Arai M, Koshihara K (1995). Autoantibody to factor VIII that has less reactivity to factor VIII/von Willebrand factor complex. Am J Hematol.

[62] Gensana M, Altisent C, Aznar JA (2001). Influence of von Willebrand factor on the reactivity of human factor VIII inhibitors with factor VIII. Haemophilia.

[63] Shi Q, Kuether EL, Schroeder JA (2012). Factor VIII inhibitors: von willebrand factor makes a difference in vitro and in vivo. J Thromb Haemostas.

[64] Lenting PJ, Neels JG, van den Berg BM (1999). The light chain of factor VIII comprises a binding site for low density lipoprotein receptor-related protein. J Biol Chem.

[65] Dasgupta S, Navarrete AM, Bayry J (2007). A role for exposed mannosylations in presentation of human therapeutic self-proteins to CD4+ T lymphocytes. Proc Natl Acad Sci U S A.

[66] Behrmann M, Pasi J, Saint-Remy JM, Kotitschke R, Kloft M (2002). Von Willebrand factor modulates factor VIII immunogenicity: comparative study of different factor VIII concentrates in a haemophilia A mouse model. Thromb Haemostas.

[67] Delignat S, Repesse Y, Navarrete AM (2012). Immunoprotective effect of von Willebrand factor towards therapeutic factor VIII in experimental haemophilia A. Haemophilia.

[68] Meeks SL, Cox CL, Healey JF (2012). A major determinant of the immunogenicity of factor VIII in a murine model is independent of its procoagulant function. Blood.

[69] Gouw SC, van der Bom JG, Auerswald G, Ettinghausen CE, Tedgard U, van den Berg HM (2007). Recombinant versus plasma-derived factor VIII products and the development of inhibitors in previously untreated patients with severe hemophilia A: the CANAL cohort study. Blood.

[70] Gouw SC, van der Bom JG, Ljung R (2013). Factor VIII products and inhibitor development in severe hemophilia A. N Eng J Med.

[71] Goudemand J, Rothschild C, Demiguel V (2006). Influence of the type of factor VIII concentrate on the incidence of factor VIII inhibitors in previously untreated patients with severe hemophilia A. Blood.

[72] Goudemand J (2007). Inhibitor development in haemophilia A: the role of von Willebrand factor/factor VIII concentrates. Haemophilia.

[73] Franchini M, Lippi G (2010). Von Willebrand factor-containing factor VIII concentrates and inhibitors in haemophilia A. A critical literature review. Thromb Haemostas.

[74] Kawasaki T, Kaida T, Arnout J, Vermylen J, Hoylaerts MF (1999). A new animal model of thrombophilia confirms that high plasma factor VIII levels are thrombogenic. Thromb Haemost.

[75] Chauhan AK, Kisucka J, Lamb CB, Bergmeier W, Wagner DD (2007). von Willebrand factor and factor VIII are independently required to form stable occlusive thrombi in injured veins. Blood.

[76] Holmberg HL, Kjalke M, Karpf D (2011). High Affinity Binding of FVIII to VWF Is Not Required for the Haemostatic Effect of FVIII In Vivo. ASH Annual Meeting Abstracts.

[77] Nyman D (1977). Interaction of collagen with the factor VIII antigen-activity - von Willebrand factor complex. Thromb Res.

[78] Cruz MA, Yuan H, Lee JR, Wise RJ, Handin RI (1995). Interaction of the von Willebrand factor (vWF) with collagen. Localization of the primary collagen-binding site by analysis of recombinant vWF a domain polypeptides. J Biol Chem.

[79] Lankhof H, van Hoeij M, Schiphorst ME (1996). A3 domain is essential for interaction of von Willebrand factor with collagen type III. Thromb Haemost.

[80] Romijn RA, Westein E, Bouma B (2003). Mapping the collagen-binding site in the von Willebrand factor-A3 domain. J Biol Chem.

[81] Nishida N, Sumikawa H, Sakakura M (2003). Collagen-binding mode of vWF-A3 domain determined by a transferred cross-saturation experiment. Nature Struc Biol.

[82] Santoro SA (1981). Adsorption of von Willebrand factor/factor VIII by the genetically distinct interstitial collagens. Thromb Res.

[83] Denis C, Baruch D, Kielty CM, Ajzenberg N, Christophe O, Meyer D (1993). Localization of von Willebrand factor binding domains to endothelial extracellular matrix and to type VI collagen. Arterioscler Thromb.

[84] Lisman T, Raynal N, Groeneveld D (2006). A single high-affinity binding site for von Willebrand factor in collagen III, identified using synthetic triple-helical peptides. Blood.

[85] Brondijk TH, Bihan D, Farndale RW, Huizinga EG (2012). Implications for collagen I chain registry from the structure of the collagen von Willebrand factor A3 domain complex. Proc Natl Acad Sci U S A.

[86] Ribba AS, Loisel I, Lavergne JM (2001). Ser968Thr mutation within the A3 domain of von Willebrand factor (VWF) in two related patients leads to a defective binding of VWF to collagen. Thromb Haemost.

[87] Riddell AF, Gomez K, Millar CM (2009). Characterization of W1745C and S1783A: 2 novel mutations causing defective collagen binding in the A3 domain of von Willebrand factor. Blood.

[88] Flood VH, Lederman CA, Wren JS (2010). Absent collagen binding in a VWF A3 domain mutant: utility of the VWF: CB in diagnosis of VWD. J Thromb Haemost.

[89] Legendre P, Navarrete AM, Rayes J (2013). Mutations in the A3 domain of Von Willebrand factor inducing combined qualitative and quantitative defects in the protein. Blood.

[90] Pareti FI, Fujimura Y, Dent JA, Holland LZ, Zimmerman TS, Ruggeri ZM (1986). Isolation and characterization of a collagen binding domain in human von Willebrand factor. J Biol Chem.

[91] Pareti FI, Niiya K, McPherson JM, Ruggeri ZM (1987). Isolation and characterization of two domains of human von Willebrand factor that interact with fibrillar collagen types I and III. J Biol Chem.

[92] Mohri H, Yoshioka A, Zimmerman TS, Ruggeri ZM (1989). Isolation of the von Willebrand factor domain interacting with platelet glycoprotein Ib, heparin, and collagen and characterization of its three distinct functional sites. J Biol Chem.

[93] Hoylaerts MF, Yamamoto H, Nuyts K, Vreys I, Deckmyn H, Vermylen J (1997). von Willebrand factor binds to native collagen VI primarily via its A1 domain. Biochem J.

[94] Bonnefoy A, Romijn RA, Vandervoort PA, Van Rompaey I, Vermylen J, Hoylaerts MF (2006). von Willebrand factor A1 domain can adequately substitute for A3 domain in recruitment of flowing platelets to collagen. J Thromb Haemost.

[95] Morales LD, Martin C, Cruz MA (2006). The interaction of von Willebrand factor-A1 domain with collagen: mutation G1324S (type 2M von Willebrand disease) impairs the conformational change in A1 domain induced by collagen. J Thromb Haemostas.

[96] Flood VH, Gill JC, Christopherson PA (2012). Critical von Willebrand factor A1 domain residues influence type VI collagen binding. J Thromb Haemostas.

[97] McKinnon TA, Nowak AA, Cutler J, Riddell AF, Laffan MA, Millar CM (2012). Characterisation of von Willebrand factor A1 domain mutants I1416N and I1416T: correlation of clinical phenotype with flow-based platelet adhesion. J Thromb Haemostas.

[98] Larsen DM, Haberichter SL, Gill JC, Shapiro AD, Flood VH (2013). Variability in platelet- and collagen-binding defects in type 2M von Willebrand disease. Haemophilia.

[99] Marx I, Lenting PJ, Adler T, Pendu R, Christophe OD, Denis CV (2008). Correction of bleeding symptoms in von Willebrand factor-deficient mice by liver-expressed von Willebrand factor mutants. Arterioscler Thromb Vasc Biol.

[100] Marx I, Christophe OD, Lenting PJ (2008). Altered thrombus formation in von Willebrand factor-deficient mice expressing von Willebrand factor variants with defective binding to collagen or GPIIbIIIa. Blood.

[101] Wu D, Vanhoorelbeke K, Cauwenberghs N (2002). Inhibition of the von Willebrand (VWF)-collagen interaction by an antihuman VWF monoclonal antibody results in abolition of in vivo arterial platelet thrombus formation in baboons. Blood.

[102] Navarrete AM, Casari C, Legendre P (2012). A murine model to characterize the antithrombotic effect of molecules targeting human von Willebrand factor. Blood.

[103] Sixma JJ, van Zanten GH, Huizinga EG (1997). Platelet adhesion to collagen: an update. Thromb Haemostas.

[104] Nuyttens BP, Thijs T, Deckmyn H, Broos K (2011). Platelet adhesion to collagen. Thromb Res.

[105] Nieswandt B, Watson SP (2003). Platelet-collagen interaction: is GPVI the central receptor?. Blood.

[106] Inoue O, Suzuki-Inoue K, Ozaki Y (2008). Redundant mechanism of platelet adhesion to laminin and collagen under flow: involvement of von Willebrand factor and glycoprotein Ib-IX-V. J Biol Chem.

[107] Schaff M, Receveur N, Bourdon C (2011). Novel function of tenascin-C, a matrix protein relevant to atherosclerosis, in platelet recruitment and activation under flow. Arterioscler Thromb Vasc Biol.

[108] Ruggeri ZM (2009). Platelet adhesion under flow. Microcirculation.

[109] Bendetowicz AV, Wise RJ, Gilbert GE (1999). Collagen-bound von Willebrand factor has reduced affinity for factor VIII. J Biol Chem.

[110] Lopez JA, Dong JF (1997). Structure and function of the glycoprotein Ib-IX-V complex. Curr Opin Hematol.

[111] Andrews RK, Shen Y, Gardiner EE, Dong JF, Lopez JA, Berndt MC (1999). The glycoprotein Ib-IX-V complex in platelet adhesion and signaling. Thromb Haemostas.

[112] Huizinga EG, Tsuji S, Romijn RA (2002). Structures of glycoprotein Ibalpha and its complex with von Willebrand factor A1 domain. Science.

[113] Dumas JJ, Kumar R, McDonagh T (2004). Crystal structure of the wild-type von Willebrand factor A1-glycoprotein Ibalpha complex reveals conformation differences with a complex bearing von Willebrand disease mutations. J Biol Chem.

[114] Ruggeri ZM, Zarpellon A, Roberts JR, Mc Clintock RA, Jing H, Mendolicchio GL (2010). Unravelling the mechanism and significance of thrombin binding to platelet glycoprotein Ib. Thromb Haemostas.

[115] Ruggeri ZM (2003). Von Willebrand factor. Curr Opin Hematol.

[116] Andrews RK, Berndt MC (2008). Platelet adhesion: a game of catch and release. J Clin Invest.

[117] Bergmeier W, Chauhan AK, Wagner DD (2008). Glycoprotein Ibalpha and von Willebrand factor in primary platelet adhesion and thrombus formation: lessons from mutant mice. Thromb Haemostas.

[118] Fressinaud E, Baruch D, Girma JP, Sakariassen KS, Baumgartner HR, Meyer D (1988). von Willebrand factor-mediated platelet adhesion to collagen involves platelet membrane glycoprotein IIb–IIIa as well as glycoprotein Ib. J Lab Clin Med.

[119] Beacham DA, Wise RJ, Turci SM, Handin RI (1992). Selective inactivation of the Arg-Gly-Asp-Ser (RGDS) binding site in von Willebrand factor by site-directed mutagenesis. J Biol Chem.

[120] Danton MC, Zaleski A, Nichols WL, Olson JD (1994). Monoclonal antibodies to platelet glycoproteins Ib and IIb/IIIa inhibit adhesion of platelets to purified solid-phase von Willebrand factor. J Lab Clin Med.

[121] Lankhof H, Wu YP, Vink T (1995). Role of the glycoprotein Ib-binding A1 repeat and the RGD sequence in platelet adhesion to human recombinant von Willebrand factor. Blood.

[122] Zanardelli S, Chion AC, Groot E (2009). A novel binding site for ADAMTS13 constitutively exposed on the surface of globular VWF. Blood.

[123] Feys HB, Anderson PJ, Vanhoorelbeke K, Majerus EM, Sadler JE (2009). Multi-step binding of ADAMTS-13 to von Willebrand factor. J Thromb Haemost.

[124] Zannettino AC, Holding CA, Diamond P (2005). Osteoprotegerin (OPG) is localized to the Weibel-Palade bodies of human vascular endothelial cells and is physically associated with von Willebrand factor. J Cell Physiol.

[125] Shahbazi S, Lenting PJ, Fribourg C, Terraube V, Denis CV, Christophe OD (2007). Characterization of the interaction between von Willebrand factor and osteoprotegerin. J Thromb Haemost.

[126] Vinholt PJ, Overgaard M, Diederichsen AC (2013). An ELISA for the quantitation of von Willebrand Factor: Osteoprotegerin complexes in plasma. Thromb Res.

[127] Saint-Lu N, Oortwijn BD, Pegon JN (2012). Identification of Galectin-1 and Galectin-3 as Novel Partners for Von Willebrand Factor. Arterioscler Thromb Vasc Biol.

[128] Turner NA, Moake J (2013). Assembly and activation of alternative complement components on endothelial cell-anchored ultra-large von Willebrand factor links complement and hemostasis-thrombosis. PloS one.

[129] Denis C, Methia N, Frenette PS (1998). A mouse model of severe von Willebrand disease: defects in hemostasis and thrombosis. Proc Natl Acad Sci U S A.

[130] Mannucci PM, Capoferri C, Canciani MT (2004). Plasma levels of von Willebrand factor regulate ADAMTS-13, its major cleaving protease. Br J Haematol.

[131] Denis CV, Christophe OD, Oortwijn BD, Lenting PJ (2008). Clearance of von Willebrand factor. Thromb Haemost.

[132] Furlan M, Robles R, Morselli B, Sandoz P, Lammle B (1999). Recovery and half-life of von Willebrand factor-cleaving protease after plasma therapy in patients with thrombotic thrombocytopenic purpura. Thromb Haemost.

[133] Cao W, Krishnaswamy S, Camire RM, Lenting PJ, Zheng XL (2008). Factor VIII accelerates proteolytic cleavage of von Willebrand factor by ADAMTS13. Proc Natl Acad Sci U S A.

[134] Franchini M, Mannucci PM (2013). Von Willebrand disease-associated angiodysplasia: a few answers, still many questions. Br J Haematol.

[135] Franchini M, Frattini F, Crestani S, Bonfanti C, Lippi G (2013). von Willebrand factor and cancer: A renewed interest. Thromb Res.

[136] Starke RD, Ferraro F, Paschalaki KE (2011). Endothelial von Willebrand factor regulates angiogenesis. Blood.

[137] Liu FT, Yang RY, Hsu DK (2012). Galectins in acute and chronic inflammation. Ann N Y Acad Sci.

[138] Le Mercier M, Fortin S, Mathieu V, Kiss R, Lefranc F (2010). Galectins and gliomas. Brain Pathol.

[139] Pi L, Shenoy AK, Liu J (2012). CCN2/CTGF regulates neovessel formation via targeting structurally conserved cystine knot motifs in multiple angiogenic regulators. FASEB J.

[140] van Breevoort D, van Agtmaal EL, Dragt BS (2012). Proteomic screen identifies IGFBP7 as a novel component of endothelial cell-specific Weibel-Palade bodies. J Proteome Res.

[141] Fressinaud E, Meyer D (1993). International survey of patients with von Willebrand disease and angiodysplasia. Thromb Haemost.

[142] Park SO, Wankhede M, Lee YJ (2009). Real-time imaging of de novo arteriovenous malformation in a mouse model of hereditary hemorrhagic telangiectasia. J Clin Invest.

[143] Castaman G, Federici AB, Tosetto A (2012). Different bleeding risk in type 2 A and 2 M Von Willebrand disease: a two-year prospective study in 107 patients. J Thromb Haemost.

[144] Vincentelli A, Susen S, Le Tourneau T (2003). Acquired von Willebrand syndrome in aortic stenosis. N Engl J Med.

[145] Slaughter MS (2010). Hematologic effects of continuous flow left ventricular assist devices. J Cardiovasc Transl Res.

[146] Giddings JC, Banning AP, Ralis H, Lewis MJ (1997). Redistribution of von Willebrand factor in porcine carotid arteries after balloon angioplasty. Arterioscler Thromb Vasc Biol.

[147] Bosmans JM, Kockx MM, Vrints CJ, Bult H, De Meyer GR, Herman AG (1997). Fibrin(ogen) and von Willebrand factor deposition are associated with intimal thickening after balloon angioplasty of the rabbit carotid artery. Arterioscler Thromb Vasc Biol.

[148] De Meyer GR, Hoylaerts MF, Kockx MM, Yamamoto H, Herman AG, Bult H (1999). Intimal deposition of functional von Willebrand factor in atherogenesis. Arterioscler Thromb Vasc Biol.

[149] Qin F, Impeduglia T, Schaffer P, Dardik H (2003). Overexpression of von Willebrand factor is an independent risk factor for pathogenesis of intimal hyperplasia: preliminary studies. J Vasc Surg.

[150] Zhang X, Meng H, Blaivas M (2012). Von Willebrand Factor permeates small vessels in CADASIL and inhibits smooth muscle gene expression. Transl Stroke Res.

[151] Li S, Wang Z, Liao Y (2010). The glycoprotein Ibalpha-von Willebrand factor interaction induces platelet apoptosis. J Thromb Haemost.

[152] Terraube V, Pendu R, Baruch D (2006). Increased metastatic potential of tumor cells in von Willebrand factor-deficient mice. J Thromb Haemost.

[153] Terraube V, Marx I, Denis CV (2007). Role of von Willebrand factor in tumor metastasis. Thromb Res.

[154] Mochizuki S, Soejima K, Shimoda M (2012). Effect of ADAM28 on Carcinoma Cell Metastasis by Cleavage of von Willebrand Factor. J Natl Cancer Inst.

[155] Pendu R, Terraube V, Christophe OD (2006). P-selectin glycoprotein ligand 1 and beta2-integrins cooperate in the adhesion of leukocytes to von Willebrand factor. Blood.

[156] Pegon JN, Kurdi M, Casari C (2012). Factor VIII and von Willebrand factor are ligands for the carbohydrate-receptor Siglec-5. Haematologica.

[157] Bernardo A, Ball C, Nolasco L, Choi H, Moake JL, Dong JF (2005). Platelets adhered to endothelial cell-bound ultra-large von Willebrand factor strings support leukocyte tethering and rolling under high shear stress. J Thromb Haemost.

[158] Petri B, Broermann A, Li H (2010). von Willebrand factor promotes leukocyte extravasation. Blood.

[159] Zhao BQ, Chauhan AK, Canault M (2009). von Willebrand factor-cleaving protease ADAMTS13 reduces ischemic brain injury in experimental stroke. Blood.

[160] Denis CV, Andre P, Saffaripour S, Wagner DD (2001). Defect in regulated secretion of P-selectin affects leukocyte recruitment in von Willebrand factor-deficient mice. Proc Natl Acad Sci U S A.

[161] Methia N, Andre P, Denis CV, Economopoulos M, Wagner DD (2001). Localized reduction of atherosclerosis in von Willebrand factor-deficient mice. Blood.

[162] Noubade R, del Rio R, McElvany B (2008). von-Willebrand factor influences blood brain barrier permeability and brain inflammation in experimental allergic encephalomyelitis. Am J Pathol.

[163] Nichols TC, Bellinger DA, Tate DA (1990). von Willebrand factor and occlusive arterial thrombosis. A study in normal and von Willebrand’s disease pigs with diet-induced hypercholesterolemia and atherosclerosis. Arteriosclerosis.

[164] van Galen KP, Tuinenburg A, Smeets EM, Schutgens RE (2012). Von Willebrand factor deficiency and atherosclerosis. Blood Rev.

[165] Lenting PJ, Pegon JN, Groot E, de Groot PG (2010). Regulation of von Willebrand factor-platelet interactions. Thromb Haemost.

[166] Siedlecki CA, Lestini BJ, Kottke-Marchant KK, Eppell SJ, Wilson DL, Marchant RE (1996). Shear-dependent changes in the three-dimensional structure of human von Willebrand factor. Blood.

[167] Schneider SW, Nuschele S, Wixforth A (2007). Shear-induced unfolding triggers adhesion of von Willebrand factor fibers. Proc Natl Acad Sci U S A.

[168] Singh I, Shankaran H, Beauharnois ME, Xiao Z, Alexandridis P, Neelamegham S (2006). Solution structure of human von Willebrand factor studied using small angle neutron scattering. J Biol Chem.

[169] Denis CV, Lenting PJ (2012). von Willebrand factor: at the crossroads of bleeding and thrombosis. International J Hematol.

[170] Zhang X, Halvorsen K, Zhang CZ, Wong WP, Springer TA (2009). Mechanoenzymatic cleavage of the ultralarge vascular protein von Willebrand factor. Science.

[171] De Ceunynck K, De Meyer SF, Vanhoorelbeke K (2013). Unwinding the von Willebrand factor strings puzzle. Blood.

[172] Fu X, Chen J, Gallagher R, Zheng Y, Chung DW, Lopez JA (2011). Shear stress-induced unfolding of VWF accelerates oxidation of key methionine residues in the A1A2A3 region. Blood.

[173] Ganderton T, Berndt MC, Chesterman CN, Hogg PJ (2007). Hypothesis for control of von Willebrand factor multimer size by intra-molecular thiol-disulphide exchange. J Thromb Haemost.

[174] Li Y, Choi H, Zhou Z (2008). Covalent regulation of ULVWF string formation and elongation on endothelial cells under flow conditions. J Thromb Haemost.

[175] Yuan H, Deng N, Zhang S (2012). The unfolded von Willebrand factor response in bloodstream: the self-association perspective. J Hematol & Oncol.

[176] Rastegarlari G, Pegon JN, Casari C (2012). Macrophage LRP1 contributes to the clearance of von Willebrand factor. Blood.

[177] Xie L, Chesterman CN, Hogg PJ (2001). Control of von Willebrand factor multimer size by thrombospondin-1. J Exp Med.

[178] Tsai HM (2009). Mechanisms of microvascular thrombosis in thrombotic thrombocytopenic purpura. Kidney international Supplement.

[179] Crawley JT, de Groot R, Xiang Y, Luken BM, Lane DA (2011). Unraveling the scissile bond: how ADAMTS13 recognizes and cleaves von Willebrand factor. Blood.

[180] Tsai HM, Lian EC (1998). Antibodies to von Willebrand factor-cleaving protease in acute thrombotic thrombocytopenic purpura. N Engl J Med.

[181] Furlan M, Robles R, Galbusera M (1998). von Willebrand factor-cleaving protease in thrombotic thrombocytopenic purpura and the hemolytic-uremic syndrome. N Engl J Med.

[182] Levy GG, Nichols WC, Lian EC (2001). Mutations in a member of the ADAMTS gene family cause thrombotic thrombocytopenic purpura. Nature.

[183] Gandhi C, Khan MM, Lentz SR, Chauhan AK (2012). ADAMTS13 reduces vascular inflammation and the development of early atherosclerosis in mice. Blood.

[184] Fujioka M, Hayakawa K, Mishima K (2010). ADAMTS13 gene deletion aggravates ischemic brain damage: a possible neuroprotective role of ADAMTS13 by ameliorating postischemic hypoperfusion. Blood.

[185] Khan MM, Motto DG, Lentz SR, Chauhan AK (2012). ADAMTS13 reduces VWF-mediated acute inflammation following focal cerebral ischemia in mice. J Thromb Haemost.

[186] De Meyer SF, Schwarz T, Deckmyn H (2010). Binding of von Willebrand factor to collagen and glycoprotein Ibalpha, but not to glycoprotein IIb/IIIa, contributes to ischemic stroke in mice--brief report. Arterioscler Thromb Vasc Biol.

